# The Evaluation of Generative AI Should Include Repetition to Assess Stability

**DOI:** 10.2196/57978

**Published:** 2024-05-06

**Authors:** Lingxuan Zhu, Weiming Mou, Chenglin Hong, Tao Yang, Yancheng Lai, Chang Qi, Anqi Lin, Jian Zhang, Peng Luo

**Affiliations:** 1 Department of Oncology Zhujiang Hospital Southern Medical University Guangzhou China; 2 Department of Urology Shanghai General Hospital Shanghai Jiao Tong University School of Medicine Shanghai China; 3 Department of Medical Oncology National Cancer Center/National Clinical Research Center for Cancer/Cancer Hospital Chinese Academy of Medical Sciences and Peking Union Medical College Beijing China; 4 Institute of Logic and Computation TU Wien Austria

**Keywords:** large language model, generative AI, ChatGPT, artificial intelligence, health care

## Abstract

The increasing interest in the potential applications of generative artificial intelligence (AI) models like ChatGPT in health care has prompted numerous studies to explore its performance in various medical contexts. However, evaluating ChatGPT poses unique challenges due to the inherent randomness in its responses. Unlike traditional AI models, ChatGPT generates different responses for the same input, making it imperative to assess its stability through repetition. This commentary highlights the importance of including repetition in the evaluation of ChatGPT to ensure the reliability of conclusions drawn from its performance. Similar to biological experiments, which often require multiple repetitions for validity, we argue that assessing generative AI models like ChatGPT demands a similar approach. Failure to acknowledge the impact of repetition can lead to biased conclusions and undermine the credibility of research findings. We urge researchers to incorporate appropriate repetition in their studies from the outset and transparently report their methods to enhance the robustness and reproducibility of findings in this rapidly evolving field.

Since OpenAI released ChatGPT-3.5, there has been a growing interest within the medical community regarding the prospective applications of this general pretrained model in health care [1-7]. Using ChatGPT as a search keyword in the PubMed database, the results show that 2075 papers discussing ChatGPT were published in 2023. As the leading journal in the field of digital medicine, JMIR Publications Inc published a total of 115 papers related to ChatGPT in the year 2023. It should be noted that this is a quick and simple search that may not comprehensively capture all relevant articles, but it provides a general reflection of the growing interest and research on ChatGPT in the medical field. For example, Gilson et al [8] explored the performance of ChatGPT on the United States Medical Licensing Examination (USMLE) step 1 and step 2 exams, discovering that ChatGPT’s performance exceeded the passing score for third-year medical students in step 1. More studies are exploring ChatGPT’s performance on other medical exams, such as the Japanese and German Medical Licensing Examinations [9,10], the Otolaryngology-Head and Neck Surgery Certification Examinations [11], and the UK Standardized Admission Tests [12]. Beyond examinations, many articles have discussed the potential applications of ChatGPT in medicine from various perspectives. Shao et al [13] examined the suitability of using ChatGPT for perioperative patient education in thoracic surgery within English and Chinese contexts. Cheng et al [14] investigated whether ChatGPT could be used to generate summaries for medical research, and Hsu et al [15] evaluated whether ChatGPT could correctly answer basic medication consultation questions. However, we would like to point out that as a relatively new technology, there are some differences in evaluating the potential application of generative artificial intelligence (AI) like ChatGPT in health care that require additional attention from researchers.

The most significant difference affecting the evaluation of ChatGPT compared to traditional AI models known to people is the randomness inherent in the responses generated by ChatGPT. Common perception holds that for a given input, an AI model should produce the same output consistently each time. However, for natural language models like ChatGPT, this is not the case. ChatGPT generates a response by predicting the next most likely word, followed by each subsequent word. The process of generating responses involves a certain degree of randomness. If you access ChatGPT using the application programming interface, you can also control the degree of randomness in the generated responses with the temperature parameter. Even with the same input, the responses provided by ChatGPT will not be the same, and sometimes may even be completely contradictory. Therefore, when evaluating ChatGPT’s performance, it is necessary to generate multiple responses to the same input and assess these responses collectively to explore ChatGPT’s performance accurately; otherwise, there is a high likelihood of drawing biased conclusions. For example, as one of the earliest studies published, Sarraju et al [4] asked the same question three times and assessed whether the three responses given by ChatGPT to the same question were consistent. As OpenAI made the ChatGPT application programming interface accessible, it became feasible to ask the same question many more times. In a recent study investigating whether ChatGPT’s peer-review conclusions are influenced by the reputation of the author’s institution, von Wedel et al [16] conducted 250 repeated experiments for each question to mitigate the effects of ChatGPT’s randomness. However, not all researchers have recognized this aspect. For instance, in a study where ChatGPT was asked to answer the American Heart Association Basic Life Support and Advanced Cardiovascular Life Support exams, they found that ChatGPT could not pass either examination [17]. However, that study only asked the question once without repeating, which means that the randomness of ChatGPT could have had an impact on the experiment, affecting the reliability of the conclusions. In another improved study, researchers acknowledged the impact of ChatGPT’s randomness, asking each question three times. Compared to earlier results, ChatGPT’s performance in this study significantly improved, and it could pass the Basic Life Support exam [18], further underscoring the importance of repetitions. Therefore, it is inappropriate to evaluate ChatGPT’s performance based on a single response if one aims to draw rigorous, scientifically meaningful conclusions. Just as biological experiments typically require three repetitions for validity, without repetition, it becomes challenging to determine whether the observed phenomenon is an inherent characteristic of the model or merely a random occurrence. Additionally, for models intended for clinical practice applications, whether for patient education, diagnosis, or support in clinical documentation writing, we hope that ChatGPT can always provide correct and harmless responses. Repetition also allows us to evaluate the model’s stability and further assess its application value. However, we noticed that many recent manuscripts we reviewed were not aware of this, thus affecting the reliability of the conclusions.

Therefore, in research on the application of generative AI like ChatGPT in health care, appropriate repetition should be included to comprehensively evaluate the model’s performance by assessing the stability of the model in the task set by the author. This should be considered from the beginning of the research. Since models like ChatGPT will continue to be upgraded, if the authors only realize the need for repetition when revising the manuscript, there will be a considerable time gap between the authors’ supplementary analysis and the original analysis. The model has likely been upgraded during this period, introducing new uncertainties into the research. Alternatively, the authors need to completely redo the analysis from scratch during the manuscript revision process, wasting time and effort. Therefore, we hope that future researchers will recognize the necessity of repeated experiments from the start and report in the manuscript how the repetition was carried out in the study [19].

## Abbreviations

AI: artificial intelligence
USMLE: United States Medical Licensing Examination
